# Association between cardiovascular health metrics and retinal ageing

**DOI:** 10.1007/s11357-023-00743-3

**Published:** 2023-03-17

**Authors:** Ruiye Chen, Jinyi Xu, Xianwen Shang, Gabriella Bulloch, Mingguang He, Wei Wang, Zhuoting Zhu

**Affiliations:** 1 Department of Ophthalmology, Guangdong Academy of Medical Sciences, Guangdong Provincial People’s Hospital, Guangzhou, China; 2 Centre for Eye Research Australia; Ophthalmology, University of Melbourne, Melbourne, Australia; 3 Ophthalmology, Department of Surgery, University of Melbourne, Melbourne, Australia; 4 State Key Laboratory of Ophthalmology, Zhongshan Ophthalmic Center, Sun Yat-sen University, Guangzhou, China

**Keywords:** Cardiovascular health metrics, Retinal age gap, Vascular ageing

## Abstract

The study aims to investigate associations between cardiovascular health (CVH) metrics and retinal ageing indexed by retinal age gap. A total of 26,354 participants from the UK Biobank study with available CVH metrics and qualified retinal imaging were included in the present analysis. CVH included 7 metrics (smoking, physical activity, diet, body mass index [BMI], total cholesterol, blood pressure [BP], blood glucose). These were summarized to classify the overall CVH as poor (0–7), intermediate (8–10) or ideal (11–14). Retinal age gap was defined as the difference between biological age predicted by fundus images and chronological age. Accelerated and non-accelerated retinal ageing was defined if retinal age gap was in the upper or lower 50% quantiles of the study population, respectively. Linear and logistic regression models estimated the association of overall CVH and each metric of CVH with retinal age gap respectively. Our results showed that in the fully adjusted model, each one-unit score increase in overall CVH was negatively associated with retinal age gap (odds ratio [OR] = 0.89, 95% confidence interval [CI]: 0.87-0.92, *P* < 0.001). Compared with poor overall CVH, people with intermediate and ideal overall CVH had significantly lower retinal age gap (OR = 0.76, 95%CI: 0.67–0.85, *P* < 0.001; OR = 0.58, 95%CI: 0.50–0.67, *P* < 0.001). Similar associations were found between overall CVH and accelerated retinal ageing. CVH metrics including smoking, BMI, BP, and blood glucose were also significantly associated with higher retinal age gap. Taken together, we found a significant and inverse dose-response association between CVH metrics and retinal age gap, indicating that maintaining healthy metrics especially smoking, BMI, BP, and blood glucose may be crucial to slow down biological ageing.

**Supplementary Information:**

The online version contains supplementary material available at 10.1007/s11357-023-00743-3.

## Introduction

Ageing has become an urgent problem worldwide, with the proportion of people over 60 years projected to rise from 12 to 20% between 2015 and 2050. This is expected to inflate rates of morbidity and mortality worldwide [1]. Heterogeneous ageing pace has been noted in various population, which leads to different health outcomes among same age populations [2]. This highlights the need for accurately quantifying ageing process.

Vascular ageing is an important paradigm of the ageing process which contributes to the development of cardiovascular diseases (CVD) which precipitate morbidity and mortality [3, 4]. The retina uniquely visualizes the microvasculature of the body and acts as a representative window of the microcirculation [5]. Our research group previously leveraged deep learning (DL) to accurately predict chronological age based on retinal fundus images in a healthy population [6]. We verified the retinal age gap, defined as the difference between retina-predicted age and chronological age, could independently predict mortality and age-related diseases [7–10] . We also found retinal age gap was significantly associated with arterial stiffness index and future risk of cardiovascular events [

Identifying variables which influence the rate of vascular ageing is important for public health campaigns and population modelling. Cardiovascular health (CVH) is a concept introduced by the American Heart Association (AHA) in 2010 to bring about awareness and attention to the effects of CVD. Overall CVH can be classified as poor (scores 0–7), intermediate (8–10), and ideal (11–14) based on smoking, physical activity, diet, body mass index (BMI), total cholesterol, blood pressure (BP), and blood glucose. Each metric is also classified as poor, intermediate, and ideal [11]. Previously, ideal overall CVH has been associated with a lower risk of cardiovascular events and CVD-related mortality [12, 13]. Therefore, these variables are considered modifiable risk factors for CVH which may assist the prevention of vascular ageing.

To the best of our knowledge, few studies have explored associations between CVH metrics and retinal ageing. Establishing an association between these may provide an opportunity for population-based retinal screening to identify individuals at-risk of accelerated ageing, and implement strategies which inhibit the precipitation of further vascular dysfunction. Therefore, we aimed to investigate the association of CVH metrics with retinal age gap, the reliable biomarker of vascular ageing, in a large-scale, population-based cohort.

## Methods

### Study design and population

This study retrieved data from the UK Biobank study, a large, prospective, population-based cohort study comprising over 500,000 adults aged 40–69 years enrolled between 2006 and 2010. Study design and protocols have been described elsewhere [14], but in brief, baseline assessments including questionnaires and physical measurements were completed at 22 assessment centers across England, Scotland, and Wales. Biological samples of blood, urine, and saliva were also collected. Information about medical events were identified through hospital admission records and death registers.

The UK Biobank was reviewed and approved by the National Information Governance Board for Health and Social Care and the National Health Service North West Multicenter Research Ethics Committee (11/NW/0382). Data used in the present study was accessed through the Biobank consortium (Application No: 62489). Since this is a publicly identified dataset, the Guangdong Provincial People’s Medical Research Ethics Committee waived the ethical requirement. This study was carried out in accordance with the Helsinki declaration, and informed consent was provided from all participants.

### Measurement and definition of CVH

According to AHA guidelines, overall CVH is based on 7 behavioral and biological metrics, including smoking, physical activity, diet, BMI, total cholesterol, BP, and blood glucose. Each metric was categorized as poor (scored as 0), intermediate (scored as 1), and ideal (scored as 2) according to the modified AHA criteria (Supplemental Table 1). We summarized the seven metrics as a CVH score ranging from 0 to 14. The overall CVH was divided into poor (0–7), intermediate (8–10), and ideal (11–14) with higher scores corresponding to better CVH.

For smoking status, current smokers were assigned to poor, past smokers to intermediate, and ideal as never having smoked. Status of physical activity was defined as poor if physical activity was <3 metabolic equivalent tasks (MET) min/week, intermediate if activity was 3–449 MET min/week, and ideal if activity was >450 MET min/week consistent to previous studies [11, 15]. For diet, poor status was defined as <1 portion per day of fresh fruit, raw vegetables, cooked fruit/vegetables, and <2 portions per week of fish; intermediate status was defined as >1 portion per day of fresh fruit, raw vegetables, cooked fruit/vegetables, or >2 portions per week of fish; ideal health was defined as >1 portion per day of each of fresh fruit, raw vegetables, cooked fruit/vegetables, and >2 portions per week of fish [16]. Body mass index was calculated as the weight of an individual in kilograms divided by their height in meters squared. Poor status was defined as BMI >30 kg/m [2]; intermediate status was defined as BMI 25–29.9 kg/m [2]; ideal health was defined as BMI <25 kg/m [2]. For total cholesterol, poor status was defined as cholesterol >6.21 mmol/L (240 mg/dL); intermediate status was defined as cholesterol 5.18–6.18 mmol/L (200–239 mg/dL), or treated cholesterol <5.18 mmol/L (200 mg/dL); ideal status was defined as untreated and <5.18 mmol/L. Blood pressure was classified as poor (BP >140/90 mmHg), intermediate (systolic BP 120-139 mmHg or diastolic BP 80–89 mmHg or treated BP <120/80 mmHg), and ideal (untreated BP <120/80 mmHg), respectively. For blood glucose, ideal health was defined as untreated fasting plasma glucose (FPG) <100 mg/dL, intermediate health as treated FPG <100 mg/dL or FPG 100–125 mg/dL, and poor health was defined as ≥126 mg/dL.

### Fundus images

Paired retinal fundus and optical coherence tomography imaging (Topcon 3D OCT 1000 Mk2, Topcon Corp, Tokyo, Japan) data were collected. A 45-degree non-mydriatic and non-stereo fundus image centered on the macular and including the optic disc was taken for each eye. The quality check process was based on a previously developed algorithm [17]. The ground truth was the manual three-level grading system labelled by two ophthalmologists (good, usable, and reject). Fundus images with good or usable quality was considered to pass the quality check. In the baseline examination from the UK Biobank study, 80,169 images of 46,969 participants from the total obtained 131,238 images of 66,500 participants passed the image quality check.

### Deep learning model for age prediction

Details on the development and validation of the DL model have been described in detail elsewhere [6]. Briefly, a total of 19,200 retinal fundus images from 11,052 healthy participants trained and validated the DL model for chronological age prediction. If available, images from both eyes were used to maximize data volume. Mean absolute error (MAE) and correlation coefficients between retina-predicted age and chronological age assessed DL model performance in the testing dataset. The retinal age predicted by the DL model accurately quantified chronological age with a correlation coefficient of 0.80 (*P* < 0.001) and MAE of 3.55 years.

### Retinal age gap

Retinal age gap was defined as the difference between the retinal age, derived from fundus images, and the chronological age. We then transformed the retinal age gap to a binary variable where the upper 50% quantile was considered to be accelerated retinal ageing, and the lower 50% quantile was considered to be non-accelerated retinal ageing.

### Covariates

Covariates in the present study included age (continuous, years), sex (male/female), ethnicity (white/others), Townsend Deprivation Index (continuous), education attainment (college or university degree/others), C-reactive protein (continuous, mg/dL), consumption of alcohol intaking status (never or ex/current drinker), history of CVD (yes/no), and diabetes (yes/no). History of CVD was determined via data linkage to hospital admission and death registry records according to the International Classification of Diseases edition 10 (ICD-10) and the International Classification of Diseases edition 9 (ICD-9). Participants with any records of self-reported or doctor-diagnosed diabetes, history of taking anti-hyperglycemic medications, use of insulin, or HbA1C ≥48 mmol/L were considered to have diabetes mellitus.

### Statistical analysis

We summarized baseline characteristics by overall CVH and retinal ageing using descriptive statistics. We reported mean and standard deviation (SD) for continuous variables or numbers and proportions for categorical variables, respectively. We compared the baseline characteristics by overall CVH using chi-square test for categorical or one-way ANOVA for continuous variables. Age, sex, and ethnicity-adjusted logistic regression models estimated odds ratio (OR) and 95% confidence interval (CI) to determine baseline characteristics that were significantly associated with retinal ageing. Covariates significantly associated with overall CVH and/or retinal age gap were added into final regression models to calculate OR. We used linear regression and logistic regression models to investigate the association between overall CVH (independent variable) and retinal age gap. Model I adjusted for age, sex, and ethnicity; Model II additionally adjusted for educational attainment, socioeconomic status and alcohol intake, C-reactive protein (CRP), history of CVD, and diabetes. We also performed analysis to investigate the associations between each CVH metric and retinal age gap. Odds ratio with their 95% CI were reported. A two-sided *P*-value of <0.05 indicated statistical significance. All analyses were performed using Stata (version 13, StataCorp, Texas, USA).

## Results

### Study sample

**Fig. 1:**
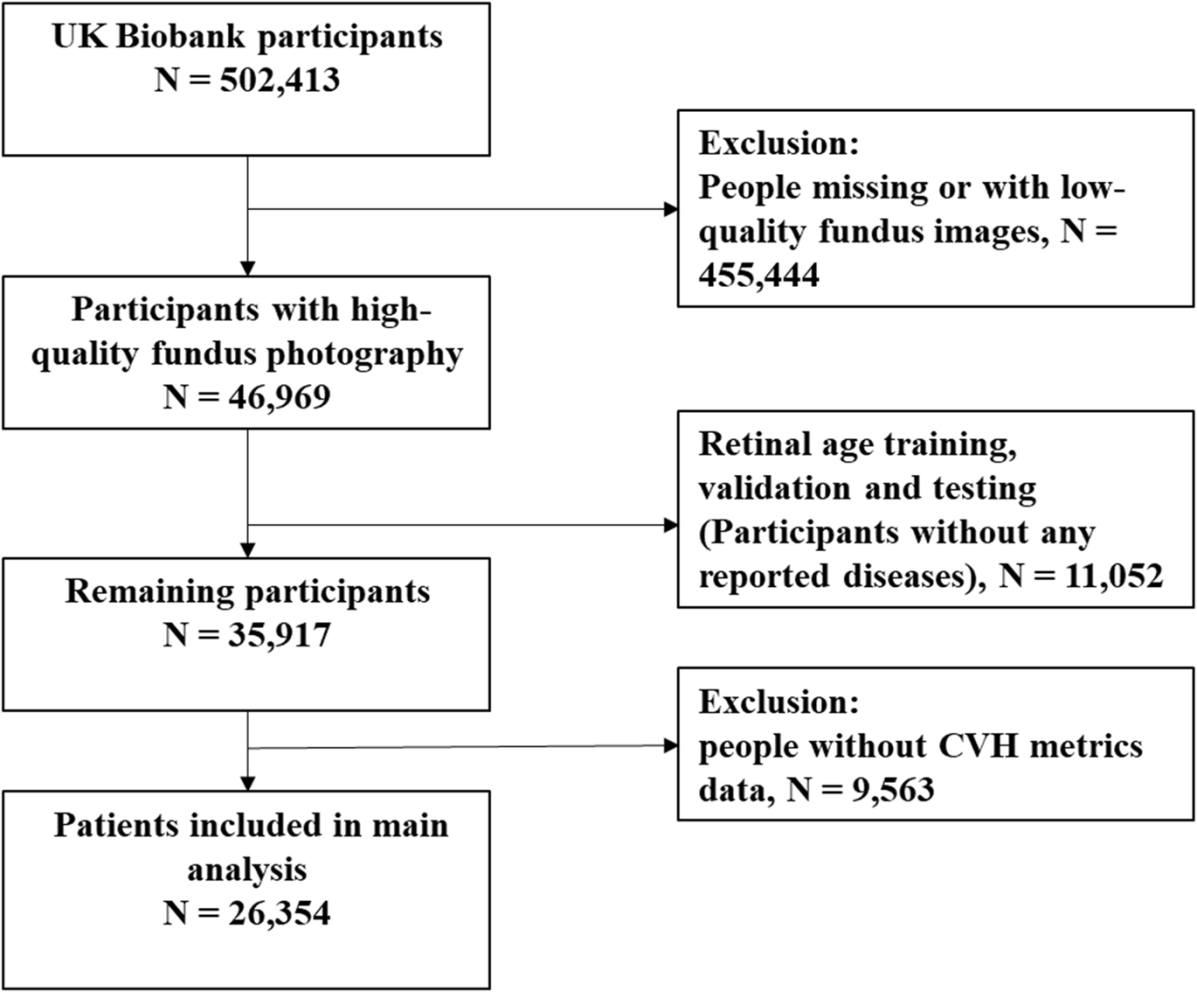
Flow chart of UK Biobank populations for study

After excluding participants whose fundus images were used for age prediction model training and validation (*N* = 11,052), and those without sufficient CVH data (*N* = 9,563), 26,354 participants (mean age 56.5 ± 8.07 years, 53.7% females) were included in the final analysis. Fundus images of the right eye estimated retinal age gap, and images of the left eye were used if images of the right eye were not available. Figure 1 shows the flow chart distribution of study population numbers. Table 1 depicts the baseline characteristics of participants stratified by overall CVH. Populations with ideal overall CVH tended to be younger, female, with better socioeconomic status, of white ethnicity, higher education level, no history of drinking alcohol, lower CRP, and no history of diabetes or CVD (*P* < 0.05).

**Table 1:** Baseline characteristics of the populations by cardiovascular health metrics

Baseline characteristics	Total	Cardiovascular health (CVH)			*P v*alue
		Poor	Intermediate	Ideal	
*N*	26,354	5886	15,180	5288	
Age, mean (SD), years	56.5 (8.07)	57.8 (7.49)	57.1 (7.94)	53.5 (8.27)	**<0.001**
Gender, no. (%)					
Female	14,152 (53.7)	2744 (46.6)	7908 (52.1)	3500 (66.2)	**<0.001**
Male	12,202 (46.3)	3142 (53.4)	7272 (47.9)	1788 (33.8)	
Deprivation index, mean (SD)	−1.15 (2.91)	−0.88 (3.01)	−1.23 (2.89)	−1.23 (2.84)	**<0.001**
Ethnicity, no. (%)					
White	24,772 (94.0)	5591 (95.0)	14,264 (94.0)	4917 (93.0)	**<0.001**
Others	1582 (6.00)	295 (5.01)	916 (6.03)	371 (7.02)	
Education level, No. (%)					
College/university	9922 (37.7)	1690 (28.7)	5699 (37.5)	2533 (47.9)	**<0.001**
Others	16,432 (62.4)	4196 (71.3)	9481 (62.5)	2755 (52.1)	
Alcohol drinking status, no. (%)					
Never	1019 (3.87)	203 (3.45)	578 (3.81)	238 (4.50)	**0.014**
Ex/current	25,320 (96.1)	5679 (96.6)	14,593 (96.2)	5048 (95.5)	
CRP, mean (SD), mg/L	2.47 (4.21)	3.35 (4.55)	2.37 (3.94)	1.78 (4.40)	**<0.001**
History of Diabetes, no. (%)					
No	24,725 (93.8)	4956 (84.2)	14,532 (95.7)	5237 (99.0)	**<0.001**
Yes	1629 (6.18)	930 (15.8)	648 (4.27)	51 (0.96)	
History of cardiovascular diseases, no. (%)					
No	24,884 (96.1)	5405 (94.5)	14,353 (96.2)	5126 (97.6)	**<0.001**
Yes	1011 (3.90)	316 (5.52)	567 (3.80)	128 (2.44)	

*SD* standard deviation, *CVH* cardiovascular health, *CRP* c-reactive protein

### Baseline characteristics associated with retinal ageing

Table 2 shows the baseline characteristics of participants stratified by classification of accelerated or non-accelerated retinal ageing. After adjusting for age, sex, and ethnicity, only socioeconomic status (OR = 1.02; 95%CI: 1.01–1.03), CRP (OR = 1.02; 95%CI: 1.01–1.02), and history of diabetes (OR = 1.58; 95%CI:1.40–1.78) were significantly associated with accelerated retinal ageing (*P* < 0.05).

**Table 2:** Baseline characteristics of the populations by retinal ageing

Baseline characteristics	Non-accelerated retinal ageing	Accelerated retinal ageing	OR (95%CI) ^a^
*N*	13,177	13,177	
Age, mean (SD), years	61.0 (6.08)	52.1 (7.30)	**0.83 (0.83–0.84)**^**b**^
Gender, no. (%)			
Female	6687 (50.8)	7465 (56.7)	1 [Reference]
Male	6490 (49.3)	5712 (43.4)	**0.85** (0.81–0.91)^**b**^
Ethnicity, no. (%)			
White	12,476 (94.7)	12,296 (93.3)	1 [Reference]
Others	701 (5.32)	881 (6.69)	**0.50 (0.44–0.57)**^**b**^
Deprivation index, mean (SD)	−1.38 (2.80)	−0.92 (3.00)	**1.02 (1.01–1.03)**
Education level, no. (%)			
College/university	4562 (34.6)	5360 (40.7)	1 [Reference]
Others	8615 (65.4)	7817 (59.3)	0.97 (0.92–1.04)
Alcohol drinking status, no. (%)			
Never	526 (3.99)	493 (3.74)	1 [Reference]
Ex/current	12,646 (96.0)	12,674 (96.3)	0.91 (0.78–1.07)
CRP, mean (SD), mg/L	2.44 (4.02)	2.50 (4.39)	**1.02 (1.01–1.02)**
History of diabetes, no. (%)			
No	12,345 (93.7)	12,380 (94.0)	1 [Reference]
Yes	832 (6.31)	797 (6.05)	**1.58 (1.40–1.78)**
History of cardiovascular diseases, no. (%)			
No	12,249 (95.0)	12,635 (97.2)	1 [Reference]
Yes	641 (4.97)	370 (2.85)	1.15 (0.98–1.33)

*SD* standard deviation, *CRP* c-reactive protein, *CI* confidence interval

^a^Adjusted for age, sex, and ethnicity

^b^*P* < 0.001

### Overall CVH and retinal age gap

After adjusting for age, gender, and ethnicity, each one-unit score increases in overall CVH was independently associated with a 13% decrease in retinal age gap (OR = 0.87, 95% CI: 0.85–0.90, *P* < 0.001) (Table 3). This finding remained significant after adjusted for further confounders (OR = 0.89, 95% CI: 0.87–0.92, *P* < 0.001). Participants with an intermediate and ideal overall CVH had significantly lower retinal age gap compared to those with a poor overall CVH (OR = 0.76, 95%CI: 0.67–0.85, *P* < 0.001; OR = 0.58, 95%CI: 0.50–0.67, *P* < 0.001; respectively). The trend between overall CVH and retinal age gap was significant (*P* < 0.001).

**Table 3:** Association between cardiovascular health and retinal age gap as a continuous outcome

	Model I		Model II	
	OR (95% CI)	*P *value	OR (95% CI)	*P *value
CVH per one unit	0.87 (0.85–0.90)	**<0.001**	0.89 (0.87–0.92)	**<0.001**
CVH				
Poor	1 [Reference]	-	1 [Reference]	-
Intermediate	0.69 (0.62–0.78)	**<0.001**	0.76 (0.67–0.85)	**<0.001**
Ideal	0.51 (0.45–0.59)	**<0.001**	0.58 (0.50–0.67)	**<0.001**
P for trending	-	**<0.001**	-	**<0.001**

Model I adjusted for age, gender, and ethnicity. Model II adjusted for age, gender, ethnicity, educational attainment, socioeconomic status and alcohol intake, c-reactive protein, history of cardiovascular disease, and diabetes

*CVH* cardiovascular health, OR odds ratio, CI confidence interval

Similar associations were identified for participants with accelerated retinal ageing, as shown in Table 4. The age, sex, and ethnicity-adjusted model showed that each one-unit increase of overall CVH was associated with a lower risk of having an accelerated retinal ageing (OR = 0.93, 95%CI: 0.92–0.95, *P* < 0.001). Participants with an intermediate and ideal overall CVH had a significantly reduced risk of accelerated retinal age compared to those with a poor overall CVH (OR = 0.79, 95%CI: 0.73–0.85, *P* < 0.001; OR = 0.72, 95%CI: 0.66–0.79, *P* < 0.001; respectively). The associations remained significant in the fully adjusted model (OR = 0.83, 95%CI: 0.77–0.90, *P* < 0.001; OR = 0.78, 95%CI: 0.71–0.86, *P* < 0.001; respectively).

**Table 4:** Association between cardiovascular health and retinal age gap as a categorical outcome

	Model I		Model II	
	OR (95% CI)	*P* value	OR (95% CI)	*P* value
CVH per one unit	0.93 (0.92–0.95)	**<0.001**	0.95 (0.93–0.96)	**<0.001**
CVH				
Poor	1 [Reference]	-	1 [Reference]	-
Intermediate	0.79 (0.73–0.85)	**<0.001**	0.83 (0.77–0.90)	**<0.001**
Ideal	0.72 (0.66–0.79)	**<0.001**	0.78 (0.71–0.86)	**<0.001**
P for trending	-	**<0.001**	-	**<0.001**

Model I adjusted for age, gender, and ethnicity. Model II adjusted for age, gender, ethnicity, educational attainment, socioeconomic status and alcohol intake, c-reactive protein, history of cardiovascular disease, and diabetes

*CVH* cardiovascular health, *OR* odds ratio, *CI* confidence interval

### CVH components and their risk with retinal ageing

The seven components which make up overall CVH were individually analyzed for their association with the retinal age gap as continuous (retinal age gap) and categorical outcomes (accelerated or non-accelerated retinal ageing) as shown in Supplementary Table 2. Compared with the poor status, ideal status of four components significantly associated with lower retinal age gap included smoking (OR = 0.73, 95%CI: 0.62–0.87), BMI (OR = 0.80, 95%CI: 0.71–0.91), BP (OR = 0.77, 95%CI: 0.66–0.89), and blood glucose (OR = 0.66, 95%CI: 0.55–0.80) (all *P* < 0.001). Similar associations were identified between ideal status of smoking (OR = 0.80, 95%CI: 0.72–0.90, *P* < 0.001), BMI (OR = 0.90, 95%CI: 0.82–0.97, *P* = 0.010), BP (OR = 0.88, 95%CI: 0.80–0.96, *P* = 0.007), and blood glucose (OR = 0.85, 95%CI: 0.75–0.95, *P* = 0.006) and accelerated retinal ageing.

## Discussion

Using the UK Biobank data, we firstly reported a significant and inverse dose-response association between CVH metrics and retinal age gap. Additionally, better CVH was associated with a lower risk of accelerated retinal ageing, and smoking, BMI, BP, and blood glucose also had significant independent associations with retinal ageing. These findings highlight the potential for retinal age gap to be used as a surrogate biomarker of vascular ageing.

Our study provides novel insights into the association between CVH metrics and currently established vascular ageing biomarkers. Several previous studies have demonstrated CVH is negatively associated with vascular biomarkers including arterial stiffness [18–20], intracranial carotid artery stenosis [21], carotid intima-media thickness [22], and carotid plaque [23, 24]. However, these studies concentrate on macrovascular dysfunction. Considering microvascular dysfunction precedes macrovascular pathology in response to cardiovascular risk factors [25–27] and retinal age is a direct output of the retinal microvasculature, our research suggests retinal age gap is likely more sensitive at tracking vascular changes allowing for earlier detection of vascular dysfunction.

Our study reveals retinal age gap could be a fast and cost-effective method to screen for premature vascular ageing in a population-based manner. In the past, poorer CVH and cardiovascular events have repeatedly been significantly associated with wider retinal venules and narrower retinal arterioles [28]. A multicenter study has demonstrated positive association between ideal CVH and retinal microvasculature measured by vessel diameters, lengths, length diameter ratio, and tortuosity [29]. Although automated segmentation exists, the interpretation of these vasculature still requires unrealistic levels of manual work. Such pitfalls inhibit the fundus images from being used widely, which highlights the advantage of retinal age as a simple and accessible solution [30].

CVH metrics including smoking, BMI, BP, and blood glucose all demonstrated significant associations with retinal ageing. Several underlying pathophysiological mechanisms may explain the associations between CVH metrics and retinal ageing. Smoking may cause microvascular dysfunction through reducing nitrogen oxide (NO) bioavailability, activation of inflammatory cascades, and tissue remodeling [31, 32]. These contribute to retinal arteriolar constriction and venular dilation [33]. Overnutrition and obesity status measured by BMI are thought to induce arteriolar and venular changes via the presence of adipokines, vasoconstrictor molecules, and decreased NO [33–36], and hypertensive states may cause vasospasm and ischemia, leading to chronic auto-regulatory vascular dysfunction and arteriosclerotic change [33, 37, 38]. Lastly, chronic hyperglycemia would trigger the activation of protein kinase C and advanced glycation end products, causing abnormal leukocyte-endothelial interactions and ultimately vascular damage in the retina [39–42]. Therefore, our findings are aligned with the current literature, which suggests retinal age gap could be a reliable indicator of vascular changes.

Our findings have several important clinical implications. First, our study explored the association between retinal age gap, a biomarker of biological age, with CVH metrics, and identified modifiable CVH metrics including smoking, BMI, BP, and blood glucose that may accelerate ageing. This highlights the promising potential of biological age for ageing research revolution and healthy ageing promotion [43]. Given the modifiable nature of CVH components, our findings emphasize the benefits of healthy lifestyles and interventions on ageing process. Second, the fast and cost-effective nature of retinal age makes it easy to implement in large-scale populations, which enables dynamic tracking and monitoring across time. This allows for the participants to have the opportunity of multiple scans, thereby facilitating individualized and real-time feedback for their proactive actions. Taken together, retinal age can be used as an accessible biological age biomarker to inform individuals about their ageing risks and important benefits of interventions for vascular health, thus reducing the overall burden of cardiovascular disease.

### Strengths and limitations

Strengths of the present study included its relatively large sample size, standardized protocol for data collection, and comprehensive adjustment of confounding factors. Despite this, some limitations should be acknowledged. Firstly, the cross-sectional nature of our study cannot infer causal relationship between CVH metrics and retinal age gap which may limit the reliability of these results to real-life circumstances. Secondly, the UK Biobank study enrolled mainly healthy and young participants which, given the geographical location, were more likely to be white ethnicity and of higher socioeconomic status. This may affect the generalizability of our results to more diverse populations [44]. However, the association between CVH metrics and retinal age gap would not be affected by the representativeness of the sample [44, 45]. Third, among the components which made up CVH, factors including physical activity and diet were subjectively measured by self-reported questionnaires, which would be subject to recall bias This may partly explain the lack of significant associations of physical activity and diet with retinal age gap in our component analyses. Finally, we were unable to fully exclude the possibility of residual confounders.

## Conclusion

In conclusion, our findings suggested a significant and inverse dose-response association between CVH metrics and retinal age gap. Maintaining health behaviors especially no smoking, good BMI, BP, and blood glucose may be crucial to reduce risk of retinal ageing. Further studies are needed to validate our findings.

## Supplementary information

ESM 1 (DOCX 29 kb)
