# Supplementary material for: Association between cardiovascular health metrics and retinal ageing
Source: GeroScience. 2023 Mar 17;45(3):1511–21. doi: 10.1007/s11357-023-00743-3 (PMC10400488; doi:10.1007/s11357-023-00743-3)
Supplement: Supplementary file 1 — (DOCX 29 kb) [file 11357_2023_743_MOESM1_ESM.docx]

Supplementary Table 1. Definition of cardiovascular health metrics.

| CVH | Poor level | Intermediate level | Ideal level |
| --- | --- | --- | --- |
| Smoking | Current smokers | Ex-smoker | Never smoker |
| Physical activity | <3 MET min/week | 3–449 MET min/week | >450 MET min/week |
| Healthy Diet | <1 portion per day of fresh fruit, raw vegetables, cooked fruit/vegetables and <2 portions per week of fish | >1 portion per day of fresh fruit, raw vegetables, cooked fruit/vegetables or >2 portions per week of fish | >1 portion per d of each of fresh fruit, raw vegetables, cooked fruit/vegetables and >2 portions per week of fish |
| Body mass index | >30 kg/m^2^ | 25-29.9 kg/m^2^ | BMI < 25 kg/m^2^ |
| Serum cholesterol | >6.21 mmol/L | 5.18-6.18 mmol/L, or treated <5.18 mmol/L | untreated <5.18 mmol/L |
| Blood pressure | SBP >140 mmHg OR DBP >90 mmHg | SBP <120 mmHg and DBP <80 mmHg treated OR SBP 120-139 OR DBP 80-89 mmHg | SBP <120 mm Hg and DBP <80 mm Hg untreated |
| Blood glucose | FPG >126 mg/dL | FPG <100 mg/dL treated OR 100-125 mg/dL | FPG <100 mg/dL untreated |

CVH = cardiovascular health; MET = metabolic equivalent tasks; BMI = body mass index; SBP= systolic blood pressure; DBP= diastolic blood pressure; FPG = fasting plasma glucose.

The AHA definitions for poor, intermediate, and ideal health were used for blood pressure, cholesterol, BMI, and physical activity; however, modified definitions were used for smoking and health diet.

Supplementary Table 2 Associations of Seven Components of CVH With Retinal Ageing.

| **Retinal ageing** | **Continuous Outcome** | | **Categorical Outcome** | |
| --- | --- | --- | --- | --- |
|  | **OR (95% CI)** | **P Value** | **OR (95% CI)** | **P Value** |
| Smoking | | | | |
| Poor | 1 [Reference] | - | 1 [Reference] | - |
| Intermediate | 0.94 (0.78-1.12) | 0.465 | 0.94 (0.83-1.05) | 0.277 |
| Ideal | 0.73 (0.62-0.87) | **<0.001** | 0.80 (0.72-0.90) | **<0.001** |
| Physical activity | | | | |
| Poor | 1 [Reference] | - | 1 [Reference] | - |
| Intermediate | 0.98 (0.68-1.42) | 0.916 | 1.01 (0.79-1.28) | 0.952 |
| Ideal | 0.73 (0.51-1.03) | 0.076 | 0.90 (0.72-1.13) | 0.373 |
| Healthy Diet | | | | |
| Poor | 1 [Reference] | - | 1 [Reference] | - |
| Intermediate | 1.22 (0.67-2.24) | 0.515 | 1.45 (0.97-2.18) | 0.071 |
| Ideal | 1.12 (0.61-2.07) | 0.715 | 1.42 (0.94-2.14) | 0.093 |
| Body mass index | | | | |
| Poor | 1 [Reference] | - | 1 [Reference] | - |
| Intermediate | 0.92 (0.81-1.03) | 0.161 | 0.95 (0.88-1.02) | 0.167 |
| Ideal | 0.80 (0.71-0.91) | **0.001** | 0.90 (0.82-0.97) | **0.010** |
| Serum cholesterol | | | | |
| Poor | 1 [Reference] | - | 1 [Reference] | - |
| Intermediate | 0.93 (0.83-1.04) | 0.184 | 0.93 (0.87-1.00) | 0.049 |
| Ideal | 0.89 (0.77-1.02) | 0.083 | 0.96 (0.88-1.05) | 0.377 |
| Blood pressure | | | | |
| Poor | 1 [Reference] | - | 1 [Reference] | - |
| Intermediate | 0.95 (0.85-1.05) | 0.286 | 0.96 (0.90-1.03) | 0.258 |
| Ideal | 0.77 (0.66-0.89) | **<0.001** | 0.88 (0.80-0.96) | **0.007** |
| Blood glucose | | | | |
| Poor | 1 [Reference] | - | 1 [Reference] | - |
| Intermediate | 0.91 (0.74-1.13) | 0.403 | 0.95 (0.83-1.09) | 0.451 |
| Ideal | 0.66 (0.55-0.80) | **<0.001** | 0.85 (0.75-0.95) | **0.006** |

CVH= cardiovascular health; OR= odds ratio; CI = confidence interval.

Model adjusted for age, gender, ethnicity, educational attainment, socioeconomic status and alcohol intake, c-reactive protein, history of cardiovascular disease and diabetes.
